# Wireless Sensor Network Optimization: Multi-Objective Paradigm

**DOI:** 10.3390/s150717572

**Published:** 2015-07-20

**Authors:** Muhammad Iqbal, Muhammad Naeem, Alagan Anpalagan, Ashfaq Ahmed, Muhammad Azam

**Affiliations:** 1 Department of Electrical Engineering, COMSATS Institute of Information Technology, Wah Campus, Wah Cantt 47040, Pakistan; E-Mails: miqbal1976@gmail.com (M.I.); muhammadnaeem@gmail.com (M.N.); ashfaq2419@gmail.com (A.A.); azamlyh@gmail.com (M.A.); 2 Department of Electrical and Computer Engineering, Ryerson University, Toronto, ON M5B 2K3, Canada

**Keywords:** algorithms, conflicting objectives, multi-objective optimization, wireless sensor network

## Abstract

Optimization problems relating to wireless sensor network planning, design, deployment and operation often give rise to multi-objective optimization formulations where multiple desirable objectives compete with each other and the decision maker has to select one of the tradeoff solutions. These multiple objectives may or may not conflict with each other. Keeping in view the nature of the application, the sensing scenario and input/output of the problem, the type of optimization problem changes. To address different nature of optimization problems relating to wireless sensor network design, deployment, operation, planing and placement, there exist a plethora of optimization solution types. We review and analyze different desirable objectives to show whether they conflict with each other, support each other or they are design dependent. We also present a generic multi-objective optimization problem relating to wireless sensor network which consists of input variables, required output, objectives and constraints. A list of constraints is also presented to give an overview of different constraints which are considered while formulating the optimization problems in wireless sensor networks. Keeping in view the multi facet coverage of this article relating to multi-objective optimization, this will open up new avenues of research in the area of multi-objective optimization relating to wireless sensor networks.

## 1. Introduction

Optimization plays a key role in wireless sensor networks. The optimization in WSNs can be broadly categorized into single and multi-objective optimization problem. In single objective optimization, the main aim of the optimizer is to minimize or maximize one objective under various constraints. Where as, in multi-objective optimization multiple objectives are simultaneously optimized. Most of the real-world problems involve multiple objectives, where all objectives need to be optimized simultaneously. This condition makes the multi-objective optimization (MOO) a challenging task and undoubtedly a very hot topic of research for theorists and engineers [1–6]. Usually, the problem formulation is done as an initial phase, where the desired scenarios are formulated as multi-objective optimization problems, and are solved by using different algorithms. The multiple objectives may or may not be conflicting, but in most of the cases, the objectives conflict with each other [7–10]. Therefore, it is very less probable to find a global optimal solution, contrary to the problems of the single objective optimization [11]. In MOO there exists multiple optimal solutions, and the decision maker has to choose the best among them, depending on the priorities of the objectives to be achieved. Depending upon the preference of the multiple objectives, the optimization problem can be tackled using various techniques [12]. The most commonly used approach is to combine multiple objectives to one figure of merit by assigning different weights to different objectives and then perform single objective optimization algorithm. Weights can be assigned to multiple conflicting objectives through direct assignment, eigenvector method, entropy method and minimal information method, *etc.* Few other commonly used multi-objective handling techniques are Min-Max, Pareto, Ranking, Goals, Preference, Gene, Sub-population, Lexicographic, Phenotype sharing function and Fuzzy [13].

WSNs have been widely adopted for monitoring purpose, e.g., to monitor the environment, habitat, greenhouse, climate, water networks [14], and personal health [15]. Similarly, WSNs have been proven a great tool for automation, e.g., home automation [16] and industrial automation [17,18] *etc.*, are few promising applications of WSNs. WSNs are composed for tiny nodes, where the nodes sense data from the environment and pass the data to the central processing unit. The nodes are usually equipped with low power, low energy and very little memory [19,20]. Due to the limited on-board resources, the designing, deployment and the operations of WSNs become challenging, while simultaneously providing the quality of service requirements [21,22]. Researchers have proposed and adopted various techniques in order to utilize the resource constrained WSNs efficiently [23–27]. For example, [26] has proposed a multi-objective hybrid optimization algorithm to solve the coverage and connectivity problem and to enhance the performance of the WSNs in terms of network life time, by joining a multi-objective on-demand algorithm employing Genetic Algorithm (GA) and a local on line algorithm. In [27], the authors have used a formulation of data aggregation problem as a mixed integer linear optimization problem, by minimizing the total power, considering the co-channel interference constraints.

Abundant literature is available where MOO has been used to solve different optimization problems relating to WSNs. This article presents an updated review of the MOO techniques being used to solve different problems relating to design, operation, deployment, placement, planning and management of WSNs. The paper provides an insight into varying degree of preferences for different conflicting objectives. Therefore, it can provide means to configure WSNs for different tradeoffs between various performance parameters depending upon the application environment of the WSN.

Highlights of the previous surveys/reviews on the topic are shown in Table 1. It can be inferred that the existing surveys do not encompass the subject completely. For example in [23] the authors have focussed the problem of node placement and surveyed different solution techniques to enhance the performance of the WSNs. The authors categorized the existing literature into dynamic and static node placement strategies. They argued that neither of the two techniques in isolation can provide the desired result. Therefore, they suggested to use a mix of static and dynamic schemes. Particle swarm optimization (PSO) techniques have been reviewed in [24] for the optimal deployment, node localization, clustering and data aggregation in wireless sensor networks (WSNs). The authors investigated PSO based techniques with respect to their suitability for WSNs and suggested how to tailor them according to the peculiar characteristics of sensor nodes. In [25], the authors have categorized various WSNs applications and reviewed different energy conservation schemes specifically, their impact on the overall performance of the specific application. They also surveyed some existing techniques based on evolutionary algorithm to achieve various trade-offs between multiple conflicting requirements for prolonging the lifetime of the WSNs.

Metaheuristic algorithms are getting popular due to their better performance in terms of convergence to the optimality and avoidance from being trapped in local optima [28]. A review is presented in [29] which elaborates application of metaheuristic algorithms to solve multi-objective optimization problems relating to data clustering in wireless sensor networks. The paper elaborates some nomenclature to highlight the aspects of clustering and depicts some important challenges to implement the technique. Biologically inspired computing for the optimization of WSNs have been reviewed in [30]. The authors have shown how the metaphoric relationship can be developed between the two systems namely, biological and non-biological. They have also shown the three stage process of ensembles design for an artificial system inspired from biological system. Therefore, the aforementioned surveys are either objective function specific or they are centered about some specific algorithms to tackle the problems relating to multi-objective optimization in WSNs. Multi-objective deployment of wireless sensor nodes has been surveyed in [31] to achieve pareto optimal front while considering multiple conflicting objectives namely, coverage, energy efficiency, lifetime and the number of sensors.

For the sake of completeness and clarity we have included some surveys relating to different multi-objective optimization algorithms and their general application in various fields. For example [2] presents an overview of methods and theory of evolutionary multi-objective optimization. Specifically, the tutorial presents basic principles of multi-objective optimization and evolutionary algorithms, and various algorithmic concepts namely, fitness assignment, diversity assurances and eliticism. The tutorial also elaborates performance of multi-objective evolutionary algorithms and highlights some issues relating to its simplified implementation. A survey of evolutionary multi-objective algorithms applied to different engineering application is provided in [3]. The authors have classified different algorithms into three different categories, namely the mehtods with a priori articulation of preferences, methods with posteriori articulation of preferences and methods with no articulation of preferences. A comprehensive survey of evolutionary based multi-objective optimization techniques is presented in [6] in a way to motivate the implementation of various techniques in the emerging technological fields. The paper elaborates each technique while focusing the relative advantages and disadvantages and the feasibility of implementation in specific application. The existing surveys [1–6,12,13,23–25,29–37] do not encompass the subject completely.

This article reviews the recent work published on multi-objective optimization algorithms applied to wireless sensor networks to achieve various trade-offs among different conflicting objectives. The existing work in this research area has been classified with respect to different network types, different applications, different solution types and different conflicting objectives. We also summarize different objectives used to formulate the multi-objective optimization problem, *i.e.*, maximization of coverage, minimization of packet error rate, maximization of network life, maximization of energy efficiency, minimization of cost, minimization of delay and maximization of throughput. We analyze the relationship between different objectives in the multi-objective formulations and present some widely used simulation tools. As an example of MOO problem, we also present a general resource allocation problem in sensor network which consists of inputs, outputs, constraints and objectives.

### Paper Organization

The paper is organized as follows: Section 2, presents a generic resource allocation problem in WSNs and Section 3 depicts classification and formulation of optimization objectives. A pictorial view of the relationship between desirable objectives is presented in Section 4 and solution approaches are classified in Section 6. In Section 5, different constraints considered while formulating MOO problems in WSNs are elaborated and Section 7, highlights existing trends of research community with respect to the research focussed on different multi-objective optimization techniques, with respect to the research focussed on different optimization formulations, with respect to the research focussed on different optimization objectives and the research emanating from different geographical areas of the world. Finally, Section 8 concludes the paper by reflecting some open challenges.

## 2. Generic Multi-Objective Optimization Problem in Wireless Sensor Networks

**Figure 1:**
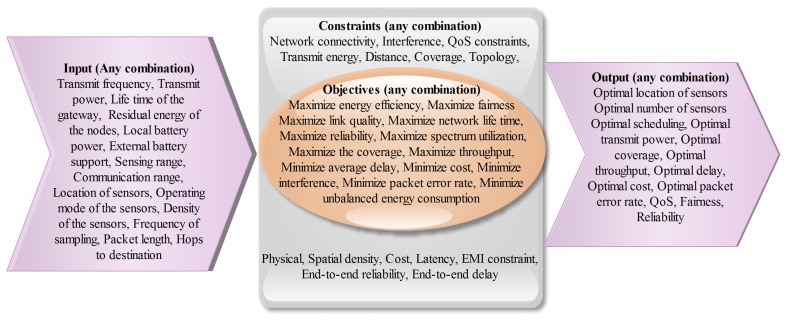
Generic multi-objective optimization problem in wireless sensor networks.

The generic multi-objective optimization problem consists of four segments: (1) inputs; (2) required output; (3) objectives; and (4) constraints. Figure 1 shows different possibilities for each part of the problem. In the generic resource allocation problem, the input parameters/decision variables are set by the network operators or the regulatory authorities. For example, selection of transmit frequency is influenced by the surrounding radio frequency environment and the regulatory rules. The selection of frequency can affect the transmission range of the sensors and ultimately many important performance parameters namely, coverage, bit error rate and delay. Increasing or decreasing the transmit power can significantly impact many desirable objectives namely, maximizing energy efficiency, link quality, network life time, reliability, coverage, cost and packet error rate. In [38], the authors have proposed an optimization formulation to maintain sensing coverage by keeping a minimum number of active sensor nodes and a small amount of energy consumption in wireless sensor network. Energy consumption has been considered in [39] by simultaneously satisfying delay and reliability through a multiobjecitve optimization algorithm. Total energy and residual energy of the nodes can also affect many performance indicators for example, coverage, throughput, network life time and packet error rate. A multi-objective formulation has been used in [40] to achieve a tradeoff solution between energy consumption and packet error rate. Location and density of the sensors determine the overall cost and the network performance in terms of observability, coverage, transmission range, reliability and energy consumption. Practical optimization problems relating to wireless sensor networks are constrained by many factors namely, network connectivity, interference, quality of service, transmit energy, coverage, topology, density, cost, latency, reliability and delay. These constrained optimization problems are expected to precipitate in optimal location of sensors, optimal number of sensors, optimal scheduling, optimal transmit power, optimal coverage, optimal throughput, optimal delay, optimal cost, optimal packet error rate, fairness and reliability. Nature of multi-objective optimization problem will change in accordance with certain input parameters, required objective function to optimize and the constraints imposed by the specific area of sensor network deployment.

## 3. Classification of Optimization Objectives

**Figure 2:**
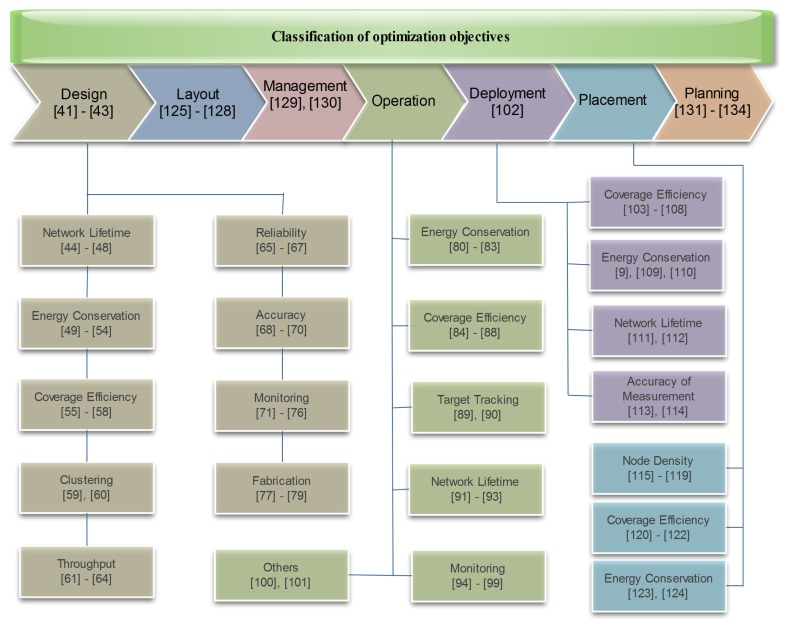
Classification of optimization objectives.

In general, many real world design problems relating to engineering are inherently characterized by the presence of multiple objectives which conflict with each other [12]. Similarly, various practical scenarios relating to efficient sensor network design, operation, placement, layout, planning and management give rise to multi-objective optimization formulations. In this section we elaborate the relevant work against each aspect of multi-objective optimization relating to WSNs as shown in Figure 2.

### 3.1. Multi-Objective Optimization Focussed on Design Related Problems in WSNs

The design of WSNs is a relatively intricate task with significant influence on various performance parameters namely, quality, cost and efficiency of real life sensor applications. One of the design goals is to maximize the lifetime of the sensor network in a way that sensors effectively monitor the region of interest and communicate the observed information to the central processing station. [41]. A multi-objective optimization approach has been proposed in [42] for the modular design architecture of QoS aware routing protocol to ensure the homogeneous depletion rate of energy. A scheme for the minimization of energy consumption has been proposed in [43] by treating the design problem of beam pattern optimization as a multi-objective formulation. Mathematical formulations of some commonly used design related objectives have been depicted in Table 2. In the following we elaborate multi-objective optimization in sensor network related to various design problems.

#### 3.1.1. Network Lifetime

Network lifetime is very critical parameter related to sensor network performance and has been tackled at various levels namely, design, operation and deployment. For example in [44], a stochastic multi-objective algorithm for WSNs has been proposed to maximize the aggregate utility and to extend the lifetime of the network. Space-based applications of wireless sensor networks are considered in [45], where authors have proposed a multi-objective formulation to address the problems of maximization of lifetime, minimization of energy consumption and maximization of the coverage. In [46], authors have formulated a multi-utility function to represent various performance metrics of the WSN and then jointly optimized the utility function and the lifetime maximization. A multi-objective routing protocol design has been proposed in [47,48] to maximize the lifetime while considering other conflicting objectives like, minimization of energy consumption, minimization of delay and secure routing.

#### 3.1.2. Energy Conservation

Wireless sensor nodes are inherently energy constrained devices. Furthermore, most of the times these devices are deployed in hard to reach areas where recharge or replacement of batteries is not possible. Therefore, energy conservation through efficient utilization of available energy helps to prolong the operation of the network. Maximization of energy conservation is one of the desirable objectives which has been addressed in various articles, for example in [49], a multi-objective evolutionary algorithm has been proposed to jointly optimize two conflicting objectives namely, maximization of energy conservation and maximizing the accuracy of spectrum sensing. Coverage performance and the energy conservation have been jointly optimized in [50] by formulating a multi-objective optimization problem and using evolutionary algorithm based decomposition approach. The authors showed that their algorithm performed better than the Non-dominated Sorting Genetic Algorithm II (NSGA-II) [50]. Design of routing algorithm has been proposed in [51–53] to optimize various conflicting objectives including energy conservation, packet delivery ratio, jitter, delay and robustness. The solution for energy conservation has also been searched in optimal cluster formation, for example in [54], a multi-objective optimization technique has been proposed to maximize energy conservation and to minimize the delay in data collection process.

#### 3.1.3. Coverage Efficiency

Coverage efficiency or coverage maximization is one of the key issues of sensor network deployment which is affected by other desirable objectives which may or may not conflict. Various multi-objective formulations have been proposed in the literature to maximize the coverage while considering other desirable objectives at the same time. For example in [55], the authors have proposed a hybrid multi-objective optimization technique for the design of wireless sensor networks to maximize the coverage and to minimize the energy consumption. Maximization of coverage, minimization of active sensor nodes and energy consumption have been simultaneously optimized in [56] by suggesting a multi-objective optimization technique. Simultaneous optimization of the coverage efficiency and energy consumption is one of the key design problems. In [57], the authors have proposed a solution inspired from the nature called, multi-objective evolutionary algorithm based on decomposition to simultaneously optimize the coverage control and energy consumption. A hybrid routing protocol design has been proposed in [58] by using a multi-objective optimization approach to improve the coverage efficiency and to reduce the energy consumption.

#### 3.1.4. Clustering

Multi-objective optimization based clustering schemes are being preferred over the single objective optimization based clustering techniques. Multi-objective optimization facilitates to consider multiple optimization criteria while formulating the clustering as an optimization problem. For example in [59], an automatic clustering technique is proposed which is based on the hybrid evolutionary algorithm immunized PSO. A multi-objective optimization based clustering algorithm has been proposed in [60] which simultaneously optimizes network life time, energy consumption, dead sensor nodes and delivery of total data packets to the base station. The authors argued that the proposed clustering algorithm based on particle swarm optimization gave better results as compared to the other existing methods.

#### 3.1.5. Throughput

Maximization of throughput is the critical issue in the design of energy constrained wireless sensor networks. Throughput optimization of energy sharing wireless sensor networks has been proposed in [61] for the design of energy sharing technique by using ultra-capacitor based energy harvesting system. Solar power sensor network design approach has been proposed in [62] to maximize the throughput in order to better utilize the solar power and to ensure fairness for all nodes across the network. The design of a cloud-integrated sensor network architecture has been proposed in [63] by using a multi-objective optimization algorithm to maximize the throughput and minimize the bandwidth and energy consumption. The design of efficient spectrum sensing and power allocation techniques have been proposed in [64] to maximize the throughput and minimize the interference.

#### 3.1.6. Reliability

A reliable and complete knowledge of some event of interest is mandatory for taking the desired decision. For example up-to-date and accurate information of current plant state is essential for plant monitoring, control and real time optimization. The accuracy and provision of different estimates of various parameters largely depend on the sensor network deployed in the plant. In [65], optimal design of wireless sensor networks for chemical plants is discussed using stochastic optimization technique for selecting the type, number and location of the nodes to achieve the required accuracy. A multi-objective optimization technique has been used in [66] to design an optimal routing protocol for maximizing the reliability, performance and efficiency.

The design of quality of service routing protocol is proposed in [67] which can accommodate different types of data traffic. The proposed routing protocol used multi-objective optimization to simultaneously optimize latency, reliability, residual energy in sensor nodes and transmission power between the nodes.

#### 3.1.7. Accuracy

In the process industry, wireless sensor networks are deployed to obtain accurate measurements of different process variables at different sampling rates. For example, in chemical and biochemical processes, temperature and pressure are measured more frequently whereas, molecular weight and concentration are measured less frequently. In [68], the authors have proposed a multi-objective algorithm to obtain a trade-off between the quality of measurement and the cost of the measurement. A trade-off between the two conflicting objectives of maximization of measurement accuracy and minimization of energy consumption has been achieved by using a lossy compression technique in [69]. The proposed design technique facilitates the node to transmit less amount of data after compression and hence can save energy during transmission. The design of intermittent fault detection in sensor nodes has been proposed in [70]. A trade-off has been obtained between the accuracy of fault detection and the detection latency by using a multi-objective optimization technique in there.

#### 3.1.8. Monitoring

Monitoring and identification of moving objects and differentiation between normal and abnormal events/states for the purpose of surveillance are popular applications of wireless sensor networks [71,72]. For example, the design of intelligent transportation system using sensor network has been proposed in [73] to detect the regions with vulnerable or dangerous drivers. A multi-objective sensor network model has been proposed in [74] for water sensor network design to monitor the water distribution system of municipalities. The proposed model focused on minimizing the volume of water from potential contamination, minimizing the expected time of detection and maximizing the probability of contamination detection. Monitoring of oceanic turbulence is the key to take preemptive measures for the safe transportation of mass and energy in the ocean and for the safety of the inhabitants along the costal cities [75]. Airfoil shear probes are the instruments to monitor and measure the turbulence in the ocean. A multi-objective optimization algorithm has been proposed in [76] to obtain the critical design parameters of the probe so as to enhance its sensitivity.

#### 3.1.9. Fabrication

Multi-objective optimization formulation has been used in designing various parameters during fabrication of biosensor to increase the detection sensitivity. In [77], a design guide for extremely sensitive photonic crystal biosensor has been proposed. The scheme facilitates the selection of grating pitch and duty based on the constraints of lithography and measurement system. Photonic sensors have the potential to replace the traditional electrical sensors due to their peculiar properties namely, small size and weight, enhanced sensitivity and immunity from electromagnetic interference [78]. A multi-objective optimization scheme has been proposed in [79] to design a wavelength division multiplexing fiber Bragg grating sensor network to simultaneously minimize the bandwidth of the optical source and the overlapping spectra.

### 3.2. Multi-Objective Optimization Focussed on Operation Related Problems in WSNs

There are lot of multi-objective optimization schemes which have been proposed for the optimal operation of wireless sensor networks. Table 3 shows some mathematical formulations of operation related objectives in WSNs. In this subsection, we classify the operation related activities into coverage efficiency, target tracking, energy consumption, monitoring, network life time, reliability and throughput. Multi-objective optimization related to aforementioned categories of the operational activities have been discussed in the following.

#### 3.2.1. Energy Conservation

Wireless sensor nodes need to increase their transmission power in order to increase signal to noise ratio and to decrease the bit error rate. On the other hand, increase in transmission power will compromise the energy conservation, minimization of interference and the life time of the network. Therefore, multi-objective optimization algorithms are used to obtain trade offs involving energy efficiency and other conflicting objectives. For example in [39], the authors investigated the effect of various parameters of energy consumption in nanosensor networks and proposed a multi-objective optimization formulation to achieve a balance between the energy consumption, delay and bit error rate. In [80], a multi-objective optimization algorithm has been proposed to maximize energy conservation and lifetime of the network by using a data aggregation route algorithm. A trade-off has been obtained between energy efficiency and end-to-end delay by using a multi-objective routing algorithm in [81]. A multi-objective optimization scheme has been used in [82] to simultaneously optimize the conflicting objectives namely, energy conservation, lifetime and coverage. The authors have used a probabilistic scheduling strategy to achieve a balance between the two conflicting objectives. A cross-layer mutli-objective approach has been used in [83] to obtain a trade-off between energy efficiency and packet loss.

#### 3.2.2. Coverage Efficiency

When the sensor nodes are deployed randomly, the number of sensors is usually more than necessary [84]. Therefore, it is not essential to operate all the nodes in active mode simultaneously. A proper sensor scheduling scheme is required to keep some nodes in sleep state and others in the active mode to help ensure coverage efficiency and energy conservation. A multi-objective optimization formulation has been suggested in [85] to optimize the conflicting objectives of coverage efficiency, life time and connectivity. The authors argued that the proposed algorithm could provide better coverage with the same level of energy conservation as compared to the others. Maintaining efficient coverage and prolonging the lifetime of wireless sensor networks is one of the important issues in WSNs. In [86], a multi-objective optimization algorithm has been proposed to get optimal coverage efficiency and prolonged network lifetime even in the presence of sensing errors. Pareto optimal solutions have been achieved in [87,88] for finding the balance between coverage efficiency and the capacity of the network.

#### 3.2.3. Target Tracking

Target tracking in the field of observation is one of the critical tasks performed by the wireless sensor network. Minimization of number of selected sensors for efficient target tracking has been modeled as multi-objective optimization problem in [89] which achieved a pareto optimal trade-off between the number of selected sensors and the accuracy of estimation. A generalized unscented Kalman filter tracking algorithm has been proposed in [90]. The proposed algorithm considered energy efficiency and target tracking performance simultaneously by using a multi-objective optimization formulation.

#### 3.2.4. Network Lifetime

For the prolonged operation of WSNs, efficient utilization of energy is one of the critical issues. In [91], a strategy is proposed for the maximization of the lifetime of the network by using a multi-objective clustering algorithm. To maximize the lifetime, the proposed algorithm controls the energy depletion of cluster heads in a way to balance their load which results in prevention of faster death of highly loaded cluster heads. A multi-objective optimization formulation has been proposed in [92] which selects the cluster head to maximize the lifetime of the network. Transmission range of the sensor node can affect the battery depletion and hence the lifetime of the network. In [93], an optimal transmission range has been searched to maximize the lifetime of the network by using an ant based heuristic algorithm.

#### 3.2.5. Monitoring

Wireless sensor networks are being used for monitoring and surveillance applications in various practical scenarios including warehouse monitoring, cargo fleet monitoring, home monitoring, human activity monitoring, health monitoring, industrial process monitoring and infrastructure monitoring [94–96]. A multi-objective optimization strategy has been proposed in [97] for dynamic monitoring of the bridge. The authors applied the proposed scheme on the dynamic monitoring of a bridge in Quzhou, China. The experimental results complemented the ideal information acquired by means of ANSYS simulation. A pervasive health monitoring system using body area sensor network has been discussed in [98], where authors have suggested an optimal resource allocation technique for sustainable power supply and guaranteeing the quality of service to support data streams. Accurate localization of sensor nodes is critical in many applications namely, remote patient monitoring, people and goods tracking, environment monitoring and wildlife habitat monitoring. In [99], a multi-objective optimization algorithm has been suggested to accurately localize the sensor nodes so as to measure data having more geographical relevance.

#### 3.2.6. Others

In addition to the popular target areas of multi-objective optimization relating to solving different operational tasks namely, coverage efficiency, network life time, target tracking and monitoring, there are several other areas which have also been considered. For example in [100], the authors have used multi-objective optimization formulation to control the green house environment by tuning the parameters of proportional integral and derivative controller. A framework has been proposed in [101] for such systems which collect potentially uncertain observations to be applied to various control actions during each sampling instant. One of the important parts of the this framework consists of fuzzy discrete event system model of sensor data collection so as to evaluate and fuse the sensor observations. The suggested technique is applied to a mobile robot which is assigned a task to follow a predefined path while avoiding any hurdle on the way.

### 3.3. Multi-Objective Optimization Focussed on Deployment Related Problems in WSNs

Wireless sensor network deployment problem encompasses the determination of positions for sensor nodes in order to achieve intended coverage, connectivity and energy efficiency while keeping the number of nodes as minimum as possible [102]. Optimal deployment of WSN guarantees sufficient quality of service, increased network life time and minimum cost [24]. Table 4 depicts mathematical formulations of some commonly used objectives related to deployment of wireless sensor network nodes. In the following, different objectives related to optimal deployment of wireless sensor networks are elaborated.

#### 3.3.1. Coverage Efficiency

Optimal deployment of sensor network has been considered in the context of various preferences namely, coverage efficiency, network life time, energy conservation, efficient monitoring and minimum node density. In [103], the authors have tackled the problem of optimal deployment while considering the conflicting objectives of maximizing the coverage efficiency and network life time simultaneously. A PSO based multi-objective optimization formulation has been proposed in [104], which have optimized the coverage efficiency and the energy consumption of ad hoc wireless sensor networks. Maximization of coverage efficiency and minimization of cost through optimal sensor network deployment has been addressed in [105], where authors have proposed a multi-objective optimization technique based of PSO. Optimal deployment of heterogeneous WSN has been considered in [106] to optimize the coverage, average number of hops and network reliability. Deployment of WSN for smart grid communication has been addressed in [107] by optimizing coverage and end-to-end latency. In [108], a technique for optimal deployment of sensor network has been proposed to optimize the coverage efficiency and lifetime of the network.

#### 3.3.2. Energy Conservation

Owing to the peculiarities of wireless sensor networks, energy conservation or energy efficiency is one of the most critical objectives and has been tackled at various levels namely, design, operation and deployment. Various deployment strategies have been proposed in the literature focusing on the maximization of energy conservation. For example in [9], the authors have proposed an optimal deployment scheme which considered minimization of net energy consumption along with other objectives including maximizing the area of coverage, maximizing the network life time, and minimizing the number of deployed sensor nodes. Energy conservation has been tackled in [109] through optimal deployment of body sensor network for the objectives of minimizing energy consumption, minimizing bandwidth and maximizing data yield. Deployment problem of relay node has been addressed in [110], where authors have proposed a multi-objective optimization formulation to minimize energy consumption and to maximize the coverage area.

#### 3.3.3. Network Lifetime

There are several research work relating to the deployment of sensor network while considering network lifetime along with other conflicting or non conflicting objectives. For example, optimal node deployment has been investigated in [111] to maximize optimal sensing coverage and network lifetime of wireless sensor networks. In [112], an optimal deployment of sensor network has been considered to simultaneously maximize network lifetime and coverage.

#### 3.3.4. Accuracy of Measurements

Accuracy of measurements from the sensing area is of paramount importance for extracting any conclusion from the observed data. Efforts have been made towards acquiring accurate information relating to the area or the phenomena under observation. For example, deployment of optimal sensor has been addressed in [113] by using a multi-objective optimization technique for simultaneous optimization of the probability of a successful search and the probability of false search. In [114] a multi-objective optimization algorithm has been suggested for optimal spectrum sensing in cognitive radio sensor network by achieving a trade off between probabilities of detection and probabilities of false alarm.

### 3.4. Multi-Objective Optimization Focused on Placement Related Problems in WSNs

Wireless sensor network placement problem encompasses the determination of positions and inter-node distance for sensor nodes in order to achieve intended coverage, connectivity and energy efficiency while keeping the number of nodes as minimum as possible. Optimal placement of WSN guarantees sufficient quality of service, increased network life time and minimum cost. In the following, different objectives relating to optimal placement of wireless sensor networks have been discussed.

(28) $$\begin{array}{cc} \min\text{imize} & \lambda \end{array}$$

(29) $$\underset{\vec{\gamma }ɛ\Gamma }{\min}{\left[f_{1}\left(\gamma \right)f_{2}\left(\gamma \right)\right]}^{T}$$

where γ⃗ is a sensor placement problem,

(30) $$\begin{array}{l} f_{w1,w2}\left(S,T\right)=w_{1}c_{\text{total}}+w_{2}t_{\text{total}}\\ \underset{S,T}{\min}f_{w1,w2}\left(S,T\right) \end{array}$$

#### 3.4.1. Node Density

Finding optimal sensor node density in the field of observation has significant influence on the quality of observation and the cost of the network. Various multi-objective optimization algorithms have been proposed in the literature to find the optimal node density along with considering other objectives that may or may not conflict with it. For example, in [115], the authors have proposed a multi-objective optimization algorithm to find the optimal quantity and location of sensor nodes for stay cable damage identification of cable-stayed bridge under uncertainty. A multi-objective heuristic localization technique for wireless sensor network has been proposed in [116] which is based on harmony search algorithm. The proposed approach is focused on minimization of squared error between the estimated and measured spacing between the nodes and the number of connectivity neighborhood constraints violated by the candidate topology. In [117], a multi-objective optimization formulation has been used to maximize the observations of the smart grid system while keeping the number of phasor measurement units as minimum as possible. The authors also considered contingency constraints and optimal allocation of these sensor devices on utility systems. Simultaneous minimization of the number of nodes and the energy consumption for wireless sensor network has been addressed in [118] by using a multi-objective optimization based on hybrid evolutionary algorithm. Optimal sensor network placement for the observation of water distribution system has been addressed by [119], where authors have proposed a multi-objective optimization algorithm to minimize the number of sensors and their optimal placement to ensure a prescribed reliability level for the network.

#### 3.4.2. Coverage Efficiency

Optimal placement of sensor network has been considered in the context of various preferences namely, coverage efficiency, network life time, energy conservation and minimum node density. In [120] a multi-objective evolutionary algorithm has been proposed for solving optimal sensor placement problem. The proposed approach has been used to maximize coverage, maximize desired connectivity level and minimize energy cost. Optimal sensor node placement in the field of interest has been addressed in [121], by utilizing a biologically inspired multi-objective optimization algorithm. The proposed algorithm searched optimal placement of sensor network to maximize the coverage and connectivity with minimum energy consumption. Simultaneous optimization of coverage efficiency and network life time has been addressed in [122] through optimal deployment of sensor nodes.

#### 3.4.3. Energy Conservation

Energy conservation is one of the critical issues due to the peculiar characteristics of the wireless sensor networks. Therefore, it has been discussed at various levels namely, design, operation, deployment and placement. A multi-objective optimization algorithm has been proposed in [123] to find the optimal placement of sensor network while simultaneously optimizing energy consumption and detection capability. Relay node placement problem in wireless sensor network has been addressed in [124] by using a multi-objective optimization formulation to search a trade-off between the average energy consumption and average coverage.

### 3.5. Multi-Objective Optimization Focussed on Layout Related Problems in WSNs

Layout of wireless sensor network deals with determining optimal location of sensor node in order to maximize the coverage, minimize energy consumption and to prolong the life time of the network. For example in [125], the authors have used a multi-objective optimization technique to solve the sensor layout problem with the objective of minimizing energy consumption and the number of nodes while considering the constraint of full coverage. Sensor layout problem has been addressed in [126] to minimize the number of sensors used while maximizing the quality of information contained in the measurement data for the identification of structural damage. A multi-objective optimization formulation has been suggested in [127] for the optimal layout of wireless sensor network. The proposed approach obtained a trade-off between maximization of the coverage and the life time of the network. Furthermore, the authors also investigated the impact of sensing range and communication range on the optimal layout. An energy efficient layout strategy has been proposed in [128] to maximize the coverage and life time of the network by using a multi-objective particle swarm optimization algorithm.

(31) $$\begin{array}{l} f_{1}\left(x\right)=\text{Length}\left(x\right)\\ f_{2}\left(x\right)=\text{Max}\left({\left\{E\left(x_{i}\right)\right\}}_{i=1}^{f_{1}\left(x\right)}\right) \end{array}$$

(32) $$\begin{array}{c} \text{Coverage}=\left[U_{i=1}^{n}R_{\text{Sensor}}^{2}\left(x_{i},y_{i}\right)/\text{Area}\right]\\ \text{Lifetime}=\underset{i=1,\ldots ,n}{\min}\left(T_{\text{failure},i}\right)/T_{\text{max}} \end{array}$$

where *E*() is the function related to consumed energy.

### 3.6. Multi-Objective Optimization Focussed on Network Management Related Problems in WSNs

Performance of wireless sensor networks can be improved by dynamically managing the settings of sensor network nodes. The sensor settings which can be manipulated namely, detection threshold, sensor selection for fusion and specific fusion rule, can influence the measurements of the sensors. To get the optimal settings of the sensor network parameters, various multi-objective optimization techniques have been proposed in the literature, for example in [129], the authors have proposed an optimal management strategy to find the appropriate settings of biometric sensors. In the proposed approach, risk is modeled as a multi-objective optimization formulation with global false acceptance rate and global false rejection rate as the two competing objectives. An optimal network management methodology has been suggested in [130] through the use of an evolutionary multi-objective optimization algorithm. The proposed management strategy is used to maximize the coverage area of the sensor field and minimize the overlapping of the area being covered by the neighboring sensors.

### 3.7. Multi-Objective Optimization Focussed on Planning Related Problems in WSNs

Optimal planing of the sensor network is a fundamental issue, both from the effective observability point of view and from the economic point of view [131,132]. Various attempts have been made to solve the optimal planning problem of wireless sensor networks, for example in [133], the authors have used a multi-objective optimization algorithm to find trade-off between hardware cost, coverage, link quality and life time of wireless sensor networks. Radio frequency identification network planning has been considered in [134] by using a multi-objective optimization algorithm to simultaneously optimize the coverage of the radio frequency tag, load balance, economic efficiency and interference.

(33) $$B\left(U_{t}\right)=\sum_{k=1}^{n}\bar{I}\left(X,Z_{k}\right)$$

## 4. Relationship between Different Desirable Objectives

**Figure 3:**
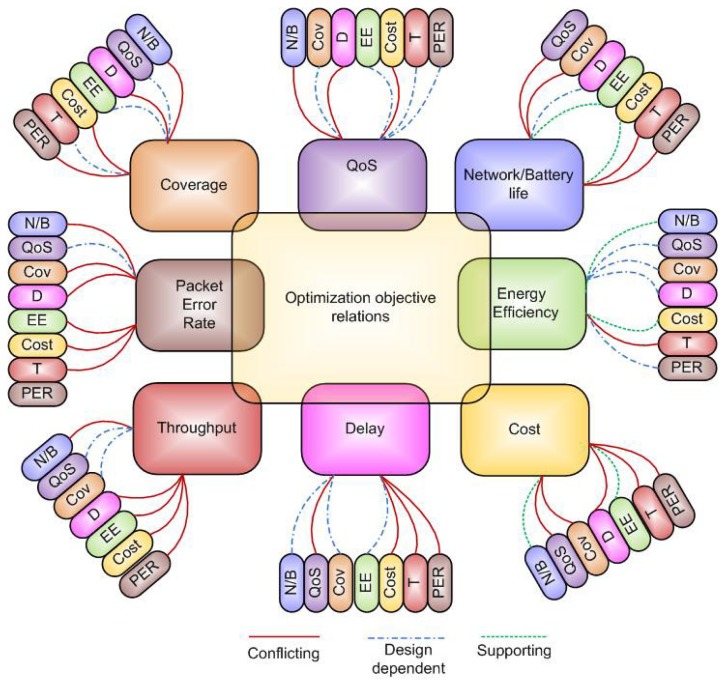
Relation between desirable objectives in wireless sensor networks (WSNs), where “N/B” = network/battery life; “QoS” = quality of service; “Cov” = coverage; “D” = delay; “Cost” = total cost of the system; “T” = throughput of the system; “EE” = energy efficiency; “PER” = packet error rate.

Most of the practical scenarios relating to wireless sensor networks are modeled as multi-objective optimization formulations where multiple desirable objectives compete with each other and the decision maker has to choose one of the tradeoff solutions. These multiple objectives may or may not conflict with each other. Figure 3 elaborates the relationship between different desirable objectives. Different objectives are connected together with lines having different pattern depending upon the relationship between objectives. Red solid line connects the two objectives which have conflicting relationship, for example, maximization of coverage conflicts with the packet error rate, delay, network/battery life time and the overall cost of the system. Whereas, the line consisting of dashes and dots connects the two objectives which have no direct relationship with each other rather they are design dependent for example, maximization of coverage has not direct relationship with the throughput, energy efficiency and the QoS. The supporting relationship between the two objectives has been shown with line consisting of dashes for example, maximization of network/battery life supports the maximization of energy efficiency and minimization of the overall cost of the system. In the following, we discuss each objective separately and its relation with other objectives.

Coverage control or coverage maximization is one of the critical research issues in wireless sensor networks and reflects the performance of the network in terms of monitoring a field of interest by properly deploying the nodes [149,150]. Coverage and lifetime of the sensor network have been jointly optimized in [86] by using a multi-objective optimization algorithm based on memetic algorithm. The sensor node deployment problem has been considered in [145] to jointly optimize the two objectives namely, maximum coverage and minimum energy consumption. Coverage, delay and energy consumption are optimized by using multi-objective optimization algorithm in [151]. The authors argued that the proposed probabilistic network model can achieve a comprehensive view of the trade-offs that result from coverage, delay and energy. Multi-objective formulation has been used in [152] to optimize the two conflicting objectives i.e., coverage and cost of WSNs. Like coverage, packet error rate is another performance parameter which also conflicts with other desirable objectives. For example in [147], three conflicting objectives namely, packet error rate, energy consumption and throughput are being considered. Packet error rate conflicts with the network/battery lifetime, as increasing the transmit power can reduce the error rate which is desirable but at the same time it will deplete the battery power more rapidly which is undesirable. Similarly, the other desirable objectives which conflict with the bit error rate are delay, energy efficiency, cost and throughput. Whereas, coverage and QoS do not have direct relationship with the error rate, rather these are design dependent. Maximization of throughput is another desirable objective which conflicts with packet error rate, cost, energy efficiency, delay and network/battery life. A multi-objective optimization framework for optimal resource allocation in cognitive radio wireless sensor networks (CRSNs) is presented in [141] to jointly optimize the conflicting objectives of maximization of throughput and minimization of total transmission power. In [143], the authors have proposed a multi-objective optimization algorithm to achieve a balance between the throughput and energy consumption of CRSNs. Minimization of end-to-end delay is desirable but it conflicts other desirable objectives including minimization of packet error rate, maximization of throughput, minimization of overall cost and QoS assurance. Whereas minimization of delay has design dependent relationship with network/battery lifetime, coverage and energy efficiency. Trade-off between end-to-end delay and energy conservation has been achieved in [153] by using heuristic optimization approach, called variance minimizing greedy minimum energy consumption forwarding protocol. A multi-objective optimization based routing scheme for wireless sensor networks has been proposed in [154] to optimize end-to-end delay, reliability, jitter, interference and energy consumption. QoS implementation is the desirable objective which conflicts with network/battery lifetime, delay and the overall cost of the wireless sensor networks. For example in [138], the authors have proposed a multi-objective routing strategy to find the trade-off between QoS and maximizing the network lifetime. A QoS aware geographic opportunistic routing protocol has been proposed as a multi-objective formulation to optimize QoS and end-to-end delay in wireless sensor networks [155].

In sensor network optimization formulations, maximization of network/battery lifetime is a desirable objective which conflicts with maximization of coverage, maximization of throughput, minimization of packet error rate and QoS. Whereas prolonging the lifetime is supported by energy efficiency and cost minimization objectives. Network/battery lifetime has no direct relationship with the minimization of delay. Energy optimized routing protocol based on clustering has been proposed in [156,157] to maximize the network lifetime and maximize the coverage. The authors have used multi-objective particle swarm optimization algorithm to find the trade-off between the two conflicting objectives. A multi-objective routing protocol has been considered in [158,159] to simultaneously optimize the two conflicting objectives namely, network lifetime and end-to-end delay. Maximization of energy efficiency conflicts with the objective of maximization of throughput whereas it supports the objectives of minimizing cost and maximizing the network/battery lifetime. For example, energy efficiency and network lifetime have been jointly optimized in [160] by considering a multi-objective hybrid routing algorithm for wireless sensor network. Whereas, energy efficiency is design dependent with respect to packet error rate, QoS, coverage and end-to-end delay. Finally, the minimization of overall cost is the ultimate objective of any network operator but it conflicts with many performance parameters of the network for example QoS, coverage maximization, delay minimization, throughput maximization and packet error rate minimization. Whereas, it is supported by the objectives of network/battery lifetime maximization and energy efficiency maximization. The problem of cost minimization and coverage maximization has been formulated as multi-objective optimization of sensor node deployment in [161]. Similarly in [162], the conflicting objectives of cost minimization and delay minimization have been jointly optimized by using a routing algorithms for hop count based forwarding in WSNs.

## 5. Constraints Employed While Formulating Optimization Problems in WSNs

**Figure 4:**
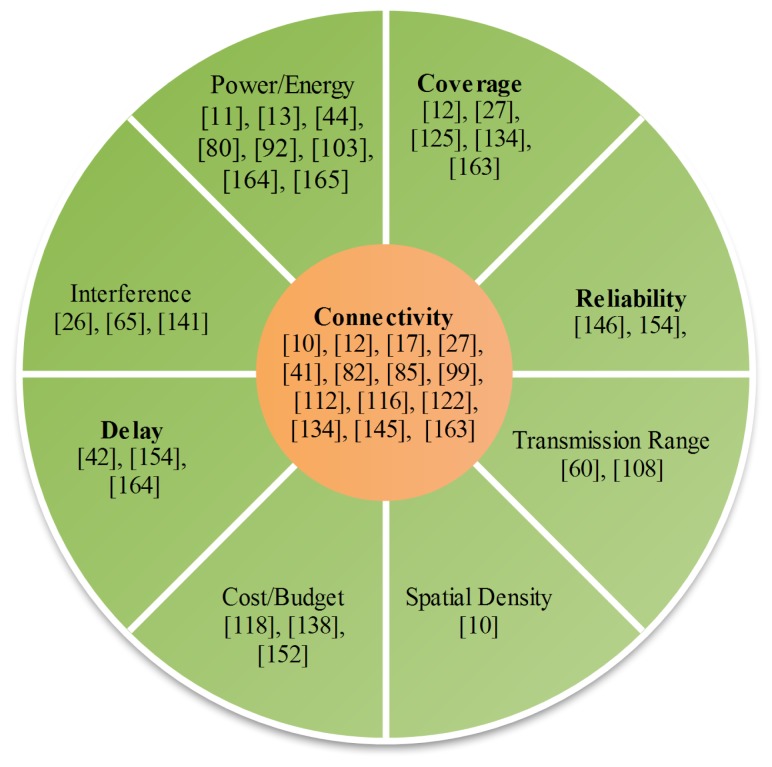
Constraints used to formulate optimization problems in WSNs.

In many practical problems, the input parameters can not be selected arbitrarily, rather they are prescribed by some physical limitations. Different configuration of the input variables can lead to different nature of optimization problem and can largely affect the output of the optimization. Figure 4 highlights various constraints which have been considered in the articles mentioned against each while formulating the optimization problem.

- For example, the constraint of ensuring connectivity between different nodes of sensor network has been considered in [85] while obtaining a trade-off between coverage rate, percentage of active sensor nodes and unbalanced energy consumption. Connectivity and coverage constraints have been considered in [134], where authors have used a multi-objective optimization to achieve a trade-off between coverage, load balance, economic efficiency and interference. In [163], the authors have used an evolutionary approach to minimize the cost of the network and maximize the system reliability while considering the constraints of coverage and connectivity.
- Energy consumption is a critical parameter which influence the overall performance of wireless sensor network and its effective lifetime. Therefore, abundant literature is available considering power or energy consumption as their design objective or considering it as the constraint while formulating the optimization problem. For example, in [164], a multi-objective optimization technique has been discussed while considering the constraint of energy consumption to maximize the prediction accuracy and minimize the latency. The constraint of energy consumption has also been considered in [165] , where the authors have proposed a technique to maximize the the coverage and efficiency of tracking the mobile targets.
- Some monitoring or measuring applications of wireless sensor networks require to provide with real time sensing capability in order to facilitate protection of those persons who are at risk to potentially harmful environments, including soldiers, first responders, and deep-sea and space explorers [166]. Some bio medical sensors require low data rate, e.g., heartbeat, blood pressure and electroencephalogram but the data may be delay sensitive and must be delivered to the main processor with in some specified time limits. In [42], a multi-objective routing algorithm has been proposed to ensure QoS for different traffic types while ensuring the delay constraints. Reliability of information transmission, interference, QoS, radio resource, coverage, topology, transmission range, number of hops, spatial density, cost and storage are few other constraints which have been considered in numerous articles while formulating the multi-objective optimization problem for the optimization of wireless networks.

## 6. Solution Types/Algorithms

**Figure 5:**
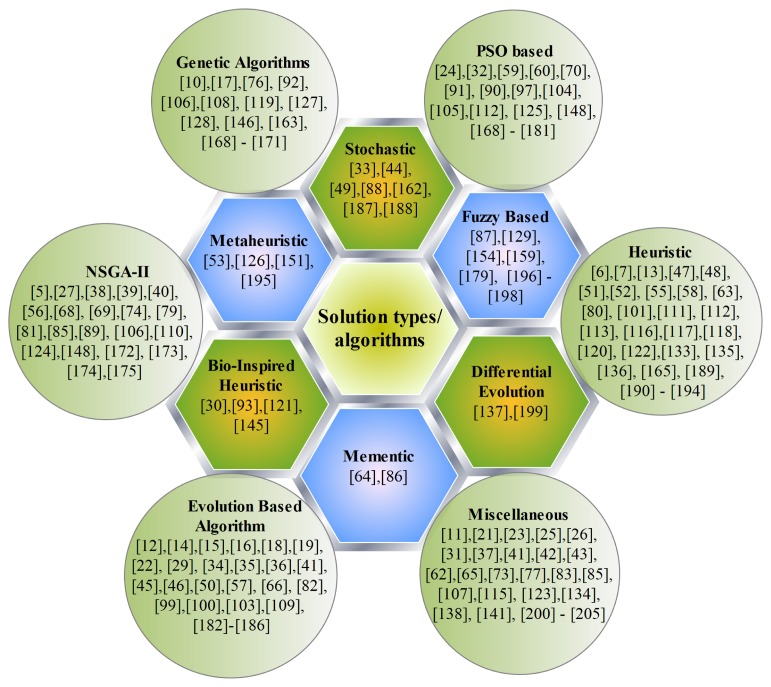
Different Types of Solution Algorithms.

A general optimization problem consists of input variables, outputs, constraints and objective function. In most of the optimization problems relating to the wireless sensor networks, these constituent parts can be combined with many different combinations giving rise to many different types of optimization problems. Therefore, no single solution algorithm exists which can provide optimal solution to different optimization problems related to wireless sensor network. Figure 5 shows the general classification of solution types to solve different multi-objective optimization problems which have been elaborated below.

### 6.1. Genetic Algorithms

Genetic Algorithms (GA) try to emulate natural evolution process by assigning a fitness value to each competing solution of the problem and employing the principle of survival of the fittest. The landmark work of [167], where genetic algorithm was successfully applied to design the sensor network which precipitated the development of several other variations of GA-based techniques. For example in [168], genetic algorithm has been proposed to solve the problem of optimal deployment of wireless sensor network for maximization of the probability of successful search of a moving target in the sensing field. Genetic algorithm has been used to solve the wireless sensor network deployment problem in [169] to maximize the coverage, minimize the number of sensors deployed, maximize the mean weightage of the sensors deployed and minimize the proximity of target to sensors. In [170], the authors have used genetic algorithm to solve the multi-objective optimization formulation used to achieve optimal deployment of sensor nodes at the port of entry for inspecting the containers in order to detect the presence of illegal cargo. Genetic algorithm based normal boundary intersection algorithm has been used in [113] to solve a multi-objective optimization problem. The problem addressed the optimization of the sensor field configuration for the detection of the moving target. A multi-objective optimization technique has been proposed in [171] for the task scheduling in wireless sensor networks. The authors have suggested to use the genetic algorithm to achieve a trade off between the makespan, efficiency of task performing and lifetime of the network.

### 6.2. Non Dominated Sorting Genetic Algorithm II

Inherently, multi-objective formulations do not result in a single solution which simultaneously optimizes all objectives. Therefore, contrary to the single objective optimization, multi-objective optimization gives a large number of alternative solutions located on or near the Pareto-optimal front. Non dominated sorting algorithm II (NSGA II) has the ability to find multiple Pareto-optimal solutions in one single run [172]. NSGA II has been opted by many researchers to solve various multi-objective optimization formulations relating to different problems of wireless sensor network. For example in [173], NSGA II has been used for the topology control. The authors argued that the Pareto-optimal front can be achieved in order to obtain low power consumption, higher robust structure and lower contention among the nodes. A compatible control algorithm has been proposed in [174] for greenhouse environment by using NSGA II. The technique focuses on finding a trade-off between the minimum energy consumption and higher control precision. In [175], the authors have used NSGA II to solve a multi-objective optimization problem relating to distributed detection in wireless sensor networks. The proposed scheme was analyzed and the simulation results showed that significant energy savings at the cost of slightly increasing the best achievable decision error probability. A multi-objective optimization approach has been suggested in [148] for the optimal deployment of wireless sensor networks. The authors have used NSGA II algorithm to solve the problem and verified the results through simulation that their proposed scheme could maintain coverage and connectivity in the given sensing area with relatively small number of sensors.

### 6.3. Particle Swarm Optimization Based Algorithms

Particle swarm optimization (PSO) was developed in 1995 [176] which was based on swarm behavior such as fish and bird schooling in nature. Due to its peculiar structure, the intelligence does not reside in the individuals rather it is distributed among a group of many individuals. PSO has gained immense popularity in recent years and has been used in several research articles to solve different optimization formulations. For example in [60], a multi-objective optimization formulation has been used to obtain energy efficient clustering and routing algorithms for wireless sensor networks. The proposed algorithm was based on particle swarm optimization approach to achieve a trade off between network life time, energy consumption, dead sensor nodes and delivery of total data packets to the base station. In [91], the authors have suggested to use a multi-tier clustering approach using cultural-based multi-objective particle swarm optimization to maximize the life time of the wireless sensor networks. A cooperative spectrum sensing technique in cognitive radio network has been proposed in [177] which exploited multi-objective hybrid invasive weed optimization and particle swarm optimization. This soft decision fusion technique was suggested to optimize the global decision threshold and weight coefficient vector was assigned to each cognitive users to facilitate maximization of detection probability and minimization of false alarm probability and overall probability of error at the same time. A dynamic sensor network management technique using multi-objective particle swarm optimization has been proposed in [178]. The output of the algorithm was the selection of sensors, threshold of the individual sensor and optimal decision fusion rule. A multi-objective optimization approach for sensor network management through fitness function design has been suggested in [179], by using a particle swarm optimization. The authors argued that the swarm can be designed to reduce run time for real-time applications as well as improving the performance of the system. In [180], a multi-objective discrete particle swarm optimization for multisensor image alignment has been proposed to obtain global best match points. The intermittent fault detection in wireless sensor networks is formulated as a multi-objective optimization problem [181]. The problem is solved by using a PSO based algorithm to achieve a trade off between inter test interval and maximum number of tests required to diagnose the node failure.

### 6.4. Evolution Based Algorithms

Evolution based multi-objective optimization algorithms use a population based approach in which more than one solution participates in an iteration and evolves a new population of solutions in each subsequent iteration [11]. These algorithms are easy to implement and do not require any derivative information. Therefore, evolution based algorithms have a wide-spread applicability and have been extensively used to solve multi-objective optimization formulations relating to wireless sensor networks. For example in [9], sensor node deployment problem has been formulated as a multi-objective optimization problem. The authors suggested to use multi-objective evolutionary algorithm to find an arrangement of sensor node to maximize the area of coverage, minimize the net energy consumption, maximize the network life time and minimize the number of deployed nodes while maintaining the desired connectivity level. An evolution based multi-objective optimization algorithm has been used in [182] for guarding a central base from enemy attacks, searching out and destroying enemy units. The authors analyzed the effectiveness of their proposed algorithm and argued that it can evolve to complete the multi-objective task, each time with the loss of one sensor. Energy problems in traditional sensor nodes can be solved by using energy harvesting micro electro-mechanical systems (EH-MEMS). Multi-objective design optimization of EH-MEMS has been suggested in [183]. The authors used evolutionary based algorithm to find the trade off between energy harvesting capability and the overall volume of the device. An evolution based approach has been used in [184] to develop a multi-objective optimization formulation for determining the optimal number of sensors, locating and setting their orientation parameters in an amorphously generated 3-D terrain. This formulation has been used to find a tradeoff between maximizing the observability of the region of interest, maximizing the stealth of the sensors, and minimizing the cost of the sensors used. In [185], an evolution based algorithm has been used to minimize energy consumption and to increase life time of wireless sensor networks based on cooperative multiple input multiple output systems. A multi-objective optimization formulation based on evolutionary algorithm has been used in [186] for maximizing coverage ratio, minimizing the number of active sensors and maximizing network life time or diminishing energy consumption.

### 6.5. Bio-Inspired Heuristic Algorithms

Bio-inspired algorithms are now among the most widely used algorithms for optimization and computational intelligence. In this subsection, we review some of the work using bio-inspired algorithms to solve the multi-objective optimization formulations in order to address different issues relating to wireless sensor networks. The sensor node placement problem has been modeled as a multi-objective optimization problem in [145], where authors have used a bio-inspired algorithm to maximize the coverage and minimize the energy consumption. A bio-inspired based algorithm has been used to solve a multi-objective optimization problem in [93] by finding the optimal transmission range in order to avoid energy hole problem in wireless sensor networks and to maximize the life time of the network. A territorial predator scent marking algorithm has been used in [121] for the optimal placement of the sensor nodes by simultaneously optimizing the coverage, connectivity and energy consumption.

### 6.6. Stochastic Algorithms

Most of the real-life problems relating to various fields require to have the optimization models and computational solution algorithms that deal with the multi-objective nature and with the stochastic behavior of the problem simultaneously [33]. For example in [162], a statistically assisted routing algorithm for hop count based forwarding in wireless sensor networks has been proposed for the minimization of cost and delay of the network. A stochastic algorithm has been proposed to solve a multi-objective optimization formulation for wireless sensor networks in [44] for maximizing aggregate utility and prolonging the network lifetime. In [187], the authors have advocated to use the stochastic optimization technique for simultaneously optimizing the squared error between the inter-node distances and the number of connectivity constraints which are not satisfied. A stochastic algorithm has been used in [188] to address the problem of tracking multiple people in a network of video sensors. The authors have proposed a multi-objective optimization strategy by combining short term feature correspondences across the sensors with long term feature dependency models. The overall objective of the algorithm was to achieve simultaneous optimization of the local similarities between features along the track for each person and the long term distribution of the features along that path.

### 6.7. Heuristic Algorithms

Heuristic algorithm is a solution approach which is based on trial-and-error to achieve reasonably accurate solutions to a complex problem in a relatively practical time. Abundant literature is available in which heuristic algorithms are used to solve the multi-objective optimization problems. For example in [55], the authors have used greedy heuristic approach to solve a hybrid multi-objective optimization for simultaneously optimizing the coverage and connectivity of wireless sensor networks. A noisy optimization problem for neuronal signaling in medical sensor-actuator networks was formulated in [189], where authors used heuristic algorithm to achieve a tradeoff between signaling latency and signaling robustness. A heuristic algorithm has been proposed in [190] to solve multi-objective optimization formulation for optimizing sensor queries. The authors have used the multi-objective technique to minimize the response time of queries and energy consumption of the networks. A large number of WSNs is usually deployed in the area under observation and each node has its own set of configurations. Every configuration affects the quality of observation significantly. Therefore, configuring the network with proper parameter is critical for the performance of the network. Since the overall network configurations are huge, exhaustive search in the configuration space is not feasible. In [191], authors used a heuristic multi-objective search method to find near optimal configurations. A multi-objective optimization formulation has been used in [192] for dynamic spectrum allocation in wireless sensor networks. The proposed technique has been solved by using heuristic algorithm to obtained a trade off between maximizing fairness, maximizing spectrum utilization, reflecting the priority among sensor data and avoiding unnecessary spectrum handoff. A heuristic algorithm has been used in [193] to solve the problem of optimum design of a dual range force sensor for obtaining high sensitivity, broad bandwidth and large measurement range. In [194], the authors have used an heuristic algorithm to solve a multi-objective optimization approach for sensor arrangement in a complex indoor environment. The optimal arrangement was achieved to maximize coverage rate, minimize interference rate, and the number of sensors.

### 6.8. Metaheuristic Algorithms

Metaheuristic algorithms generally perform better than simple heuristics. Any metaheuristic algorithm consists of two components, namely selection of the best solutions and randomization. The selection of best ensures that the solutions will tend to converge to the optimality where as randomization helps avoid the solutions being trapped at the local optima [28]. Lot of literature is available where authors have used metaheuristic algorithms to solve the multi-objective optimization relating to wireless sensor networks. For example in [195], a metaheuristic algorithm has been proposed to solve multi-objective optimization problem relating to multi-radio wireless mesh networks. Metaheuristic algorithm has been used in [151] to solve a multi-objective optimization framework for achieving optimal performance of wireless ad hoc networks in terms of reliability, delay and energy spent. In [53], the authors used metaheuristic algorithm to solve a multi-objective optimization framework for routing in wireless ad hoc networks. The algorithm achieved a trade off between delay, robustness and energy consumption. Sensor network layout problem has been formulated as a multi-objective optimization formulation in [125], where authors have suggested to use metaheuristic algorithm to solve this problem.

### 6.9. Fuzzy Logic Based Algorithms

Fuzzy logic is a mathematical discipline developed to present human reasoning in rigorous mathematical notation. Unlike classical reasoning where a proposition is either true or false, fuzzy logic establishes approximate truth value of a proposition based on linguistic variables and inference rules [196]. A common approach to deal with multi-objective optimization problems is to use weighted sum based cost function which usually is not sufficient to reach the desired solution. Fuzzy logic uses a fuzzy aggregation operator, namely the ordered weighted averaging [197] as an alternative to weighted sum approach for dealing with the multi-objective cost function. Fuzzy random variables are used to represent both fuzziness and the randomness of the objectives and constraints in routing optimization model introduced in [154]. The proposed model was used to discover the optimal routes, which were the tradeoff among the multiple objectives of delay, reliability, energy, latency, jitter, communication interference and energy balance. The authors argued that the proposed method fully utilized the advantages of Pareto optimal solution with the single run of the algorithm. In [159], the authors have used a fuzzy logic based algorithm to solve a multi-objective routing problem to simultaneously optimize lifetime and source to sink delay in wireless sensor network. A fuzzy based thermal management strategy has been proposed in [198] to control the temperature of a 3-D stacked system integrating cores, memories, sensors and radio frequency devices. The efficiency of the such a microprocessor system-on-chips is affected by the temperature. The proposed algorithm used a fuzzy controller to efficiently control the temperature without compromising the other performance parameters.

### 6.10. Differential Evolution Based Algorithms

Differential evolution is a solution algorithm to address multi-objective optimization problems. This technique is based on trial-and-error approach for finding different tradeoffs while dealing with multiple conflicting objectives. For example in [136], a differential evolution based clustering algorithm has been proposed for wireless sensor networks to increase the lifetime of the network. The authors investigated the proposed algorithm and found that its performance was better than the other existing protocols in terms of network life, number of dead sensor nodes, energy consumption and convergence rate of the algorithm. A multi-objective differential evolution algorithm has been used in [199] for the automatic clustering with application to micro-array data analysis. The authors compared the performance of their proposed algorithm with other state of the art algorithms specially NSGA II and found that the performance of their proposed scheme was better than others.

### 6.11. Memetic Algorithms

Memetic algorithms are computational intelligence structures which also exploit trial-and-error strategy to find the Pareto optimal solution set. A multi-objective coverage optimization scheme based on memetic algorithm has been proposed in [86] for the optimization of coverage. The authors argued that the algorithm could achieve optimal deployment of network coverage while considering coverage degree, node utilization, and node residual energy. A multi-objective memetic algorithm has been presented in [64] for a joint spectrum sensing and power allocation problem in a multichannel, multi-user cognitive wireless network. Efficient spectrum sensing and power allocation scheme was designed to maximize the throughput of secondary users and minimize the interference to primary users in a cognitive sensor network paradigm.

### 6.12. Miscellaneous Algorithms

In addition to the more commonly used algorithms as discussed in the preceding subsections, there are many other algorithms which have been used to address different multi-objective optimization problems relating to wireless sensor networks. For example in [8], a goal programming approach has been used to solve a multi-objective optimization formulation for maximizing network life time and maximizing the throughput for multimedia wireless sensor networks. In [27], Lagrangean relaxation technique has been used to solve a multi-objective optimization based channel constrained data aggregation routing algorithm in multi radio wireless sensor networks to minimize the total transmission. Lexicographic optimization based on greedy approach has been used in [42] to customize the QoS services for each traffic category in body wireless sensor networks. Game theoretic approach has been proposed in [200] to address a multi-objective optimization formulation for maximizing the success ratio of key management service and minimizing the nodes's cost of security and energy. In [201], fast Lipschitz algorithm has been suggested to simultaneously optimize different conflicting objectives in wireless sensor networks. Interval programming has been proposed in [202] to solve the multi-objective optimization problem relating to sensor network deployment in the marine vehicles. A Bayesian approach has been proposed in [203] to solve the multi-objective optimization problem relating to the structural health monitoring sensor network. The proposed algorithm could successfully obtain a trade off between the cost of the and the accuracy of observation. In [204], a Bayesian approach has been suggested for optimizing decentralized detection networks. An artificial intelligence based algorithm has been proposed in [205] to solve a multi-objective optimization formulation relating to route planning of intelligent transport system employing wireless sensor network.

## 7. Multi-Objective Optimization in Wireless Sensor Networks: Trends

To show the interest of the research community in the field of multi-objective optimization for wireless sensor networks, we have categorized this trend into different dimensions.

(1) Focus of research with respect to multi-objective optimization algorithms.
(2) Focus of research with respect to optimization objectives.
(3) Focus of research with respect to nature of optimization problem.

### 7.1. Focus of Research with Respect to Multi-Objective Optimization Algorithms

**Figure 6:**
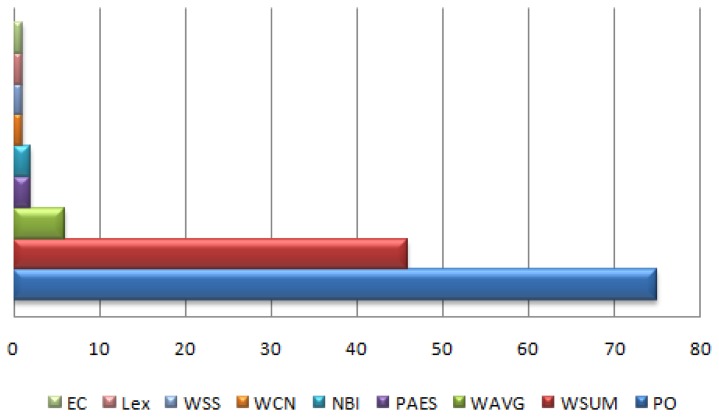
Trend of research community w.r.t. multi-objective optimization techniques. Where, EC = Epsilon constrained, Lex = Lexicographic, WSS = Weighted sum of square, WCN = Weighted chevyshev norm, NBI = Normal boundary intersection, PAES = Pareto archived evolution strategy, WAVG = Weighted average, WSUM = Weighted sum and PO = Pareto optimal.

Contrary to the single objective optimization, a solution to a multi-objective problem is a concept rather than a definition [3]. Therefore, there is usually no single global solution, and it is therefore necessary to find a set of solutions satisfying the optimality conditions. Pareto optimal solutions consist of solutions that are not dominated by any other solutions. A solution *X* is said to dominate *Y* if *X* is better or equal to *Y* in all attributes, and strictly better in at least one attribute [12]. Therefore, Pareto optimal solutions provide different trade-off scenarios where none is better than the other and the decision maker chooses one according to the preferences or specific requirements. Due to the its characteristics to achieve different trade-off solutions, Pareto optimal solution approaches are being preferred which is evident from Figure 6. In more than half of the articles in the literature, Pareto optimal approach has been used to solve the multi-objective optimization problems. Other commonly used technique is the weighted sum approach. It scalarizes a set of objectives into a single objective by assigning different weights to each objective. Conceptually this method is simplest and also widely used but it is affected by the selection of different weights. The selection of weights depends on the preference of each objective which is decided by the decision maker [11]. Therefore, the outcome of the approach is highly sensitive to the choice of the weights. There are few other less commonly used approaches namely, weighted average, Pareto archived evolution strategy, normal boundary intersection, weighted Chevyshev norm, weighted sum of square, lexicographic and epsilon constrained.

### 7.2. Focus of Research with Respect to Optimization Objectives

**Figure 7:**
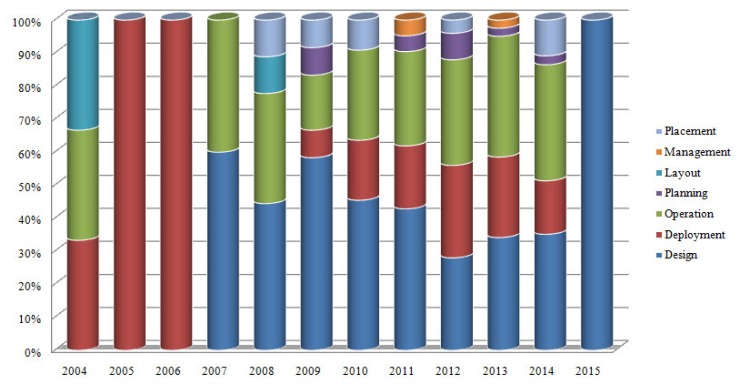
Trend of research community w.r.t. optimization objectives.

Optimization problems relating to wireless sensor networks can be broadly categorized as design optimization, deployment optimization, optimal operation, optimal planning, optimal layout, optimal management and optimal placement. Figure 7 shows distribution of articles corresponding to the aforementioned optimization objectives. Research community is predominantly inclined towards tackling the issues of design, deployment and operation related optimization problems. For example, optimal design of data forwarding protocol has been proposed in [137] to minimize energy consumption, uniform battery power depletion and minimize delay. Sensor network deployment problem has been considered in [206] with the objectives of coverage maximization, satisfaction of detection threshold and energy minimization. A coverage control strategy has been proposed in [140] for solving the conflicting problems of energy consumption, equilibrium energy and network coverage in wireless sensor networks. Less frequently tackled problems are related to planning, layout, management and placement.

### 7.3. Focus of Research with Respect to Nature of Optimization Problem

**Figure 8:**
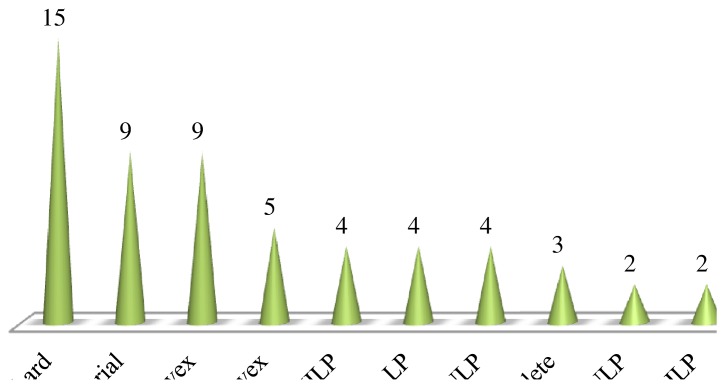
Trend of research community w.r.t. nature of Multi-objective Optimization (MOO) formulations.

In various applications of wireless sensor networks, the desirable objectives including but not limited to maximization of coverage, maximization of battery life, maximization of energy efficiency, minimization of cost, minimization of delay, maximization of throughput and minimization of packet error rate are formulated by using different optimization formulations. Different practical scenarios related to optimization give rise to different nature of optimization problem. Figure 8 shows a glimpse of the trend relating to different optimization formulations. It is evident that most of the desirable scenarios culminate in NP-Hard optimization formulations. For example in [207], optimization of connectivity, coverage, cost, network lifetime and service quality has been formulated as NP-Hard optimization problem. The problem of optimal channel assignment to maximize the throughput, improve fairness and handoff experience of the users have been formulated as NP-Hard problem in [195]. The other commonly used optimization formulations are combinatorial, non-convex, convex, mixed-integer linear programming, linear programming, non-linear programming, NP-Complete, mixed-integer non-linear programming, integer linear programming and concave.

## 8. Conclusions and Future Work

Optimization problems relating to wireless sensor network planning, design, deployment and operation often give rise to multi-objective optimization formulations where multiple desirable objectives compete with each other and the decision maker has to select one of the tradeoff solutions. These multiple objectives may conflict with each other. For example, maximization of coverage conflicts with the packet error rate, delay, network/battery life time and the overall cost of the system. Whereas in some cases, there exist multiple objectives having no direct relationship with each other, rather they are design dependent; e.g., maximization of coverage has no direct relationship with the throughput, energy efficiency and the QoS. On the other hand, some objectives support each other; e.g., maximization of network/battery life supports the maximization of energy efficiency and minimization of the overall cost of the system. Keeping in view the nature of application, the sensing scenario, input and output of the problem, the type of optimization problem changes. To address different nature of optimization problems relating to wireless sensor network design, deployment, operation, planing and placement, there exist a plethora of optimization solution types.

Due to resource constraints of wireless sensor networks, optimization method that requires relatively less memory and computational power, and produces acceptable results is highly desirable in view of implementing it on each sensor node. This article presented a contemporary review of multi-objective optimization techniques being used to solve different problems relating to design, operation, deployment, placement, planning and management of wireless sensor networks. We analyzed the existing literature to show the trend of the research community with respect to multi-objective optimization algorithms, nature of optimization problems, year-wise optimization objectives and with respect to research emanating from different geographical areas. We also presented a generic resource allocation problem in wireless sensor networks which consists of input variables, required output, objectives and constraints. A list of constraints are also presented to give an overview of different constraints which are considered while formulating the optimization problem in wireless sensor networks. Finally, the article classified different solution algorithms being used to solve the optimization problems relating to wireless sensor networks.

Keeping in view the multi facet coverage of this article relating to multi-objective optimization, it can be expected that this article will open up new avenues of research in the area of multi-objective optimization relating to wireless sensor networks. For example, efficient wireless charging is an open challenge in wireless sensor networks in order to efficiently charge the motes and to prolong the network life time. Similarly, adopting the renewable energy sources to provide adequate power to the motes can be another challenging task. Prolonged life time coupled with the enhanced processing power can also be formulated as a multi-objective optimization problem. Similarly, simultaneous solution of security and energy issues also become a multi-objective task. Therefore, a multi-objective optimization framework can be developed to jointly optimize the security, power, lifetime and the onboard processing capability.

## Figures and Tables

**Table 1:** Existing reviews/surveys relating to multi-objective optimization in wireless sensor networks.

Ref.	Review Type			Optimization Algorithms		

	Technology Specific	Objective Specific	Generic	EA	Heuristic	Other
[1]				✓		
[2]				✓		
[3]						✓
[4]				✓		
[5]				✓		
[6]				✓		
[12]				✓		
[13]				✓		
[23]	✓	✓				✓
[24]	✓			✓		
[25]	✓	✓		✓		
[29]	✓	✓			✓	
[30]	✓	✓		✓		
[31]	✓	✓		✓		
[32]				✓		
[33]						✓
[34]				✓		
[35]		✓		✓		
[36]				✓		
[37]				✓		

**Table 2:** Design related objectives in wireless sensor networks.

**Ref.**	**Objectives**	**Equation**	**Details**
[38]	Energy consumption, transmission radius, coverage area	(1) $$\begin{array}{c} \frac{A_{area}\left(N\prime \right)}{A_{s}}\\ 1-\frac{\left|N\prime \right|}{\left|N\right|}\\ -\frac{\sum_{i=1}^{n}r_{i}^{2}}{A_{area}} \end{array}$$	Multi-objective function.Objective 1 is the coverage rate of sensor set *N′*.Objective 2 is financial cost of the sensor set *N′* and Objective 3 is coverage energy consumption of the sensor set *N′. A_s_* represents the total size of target area; *A_area_* represents the monitoring size of the sensor set *N′*; $r_{i}^{2}$ represents the sensing radius of node *n_i_.*
[42]	Customized QoS services for each traffic category	(2) $\max\text{imize}\frac{\text{dist}\left(i,s\right)-\text{dist}\left(j,s\right)}{\text{dist}\left(i,s\right)},\frac{e_{\text{res}\left(j\right)}}{e_{\text{init}}}$	Where *dist*(*a*, *b*) denotes geometric distance between nodes *a* and *b*; *e_res_*_(_*_j_*_)_ denotes the residual energy level of the neighbor nodes *j* of *i*; *e_init_* denotes the initial energy level.
[45]	Energy consumption, system lifetime, coverage	(3) $$\begin{array}{l} \min f_{1}\left(P_{t},T,N\right)=\frac{1}{\eta }\overset{N}{\underset{i=1}{\text{∑}}}\left(P_{ti}+\alpha_{ci}\right)T_{i}\\ \min f_{2}\left(P_{t},T,N\right)=\left|\tau^{*}-\tau_{sys}\right|\\ \min f_{3}\left(N\right)=1-\vartheta_{c}\\ \min f_{4}\left(N\right)=N \end{array}$$	Multi-objective function. Objective 1: Optimization of energy consumption. Objective 2: optimization of system lifetime. Objective 3: coverage optimization problem. Objective 4: Optimization of the participating number of satellites. *P_ti_* and *T_i_* denotes optimal transmission power and transmission duration of node *i*, respectively; *α_ci_* represents the equivalent circuit power consumption; *N* represents number of active satellite sensing nodes at any given time instant; *η* represents the efficiency of the power amplifier; τ* represents desired system lifetime; *τ_sys_* represents , the system lifetime of a cluster networked system; *θ_c_* represents the coverage.
[49]	Energy consumption and spectrum sensing performance	(4) $\min F\left(X\right)={\left(C_{T},Q_{f},\left(-Q_{d}\right)\right)}^{T}$	Where *X* = (*μ*, λ1, λ2), where λ1 and λ2 are censoring thresholds, and *μ* represents the probability that a node is turned off; *C_T_* denotes the average energy consumption of the entire cognitive radio; *Q_D_* denotes the global probability of spectrum sensing; *Q_f_* denotes global probability of false alarm; *T* denotes samples during one sampling process.
[50]	Coverage preservation and energy conservation	(5) $\mathrm{maximize}\;F\left(x\right)={\left(f_{1}\left(x\right),\ldots \ldots ,f_{n}\left(x\right)\right)}^{T}$	Where *x* is the decision variable vector. In general, *f*_1_, ⋯ , *f_n_* are in conflict with each other, and then finding the optimum can be interpreted as finding a good trade-off between all *f*_1_, ⋯, *f_n_* of *F*.
[135]	Energy efficiency, packet error rate, average delay	(6) $$\begin{array}{cc} \mathrm{maximize}\; & \Phi .\mu \end{array}$$	Where *μ* denotes the optimal weights vector; Φ denotes the real time vector.
[136]	Optimum structure of heat exchanger	(7) $\max:I=\frac{1000R^{2}}{\bar{\Delta P}}$	The internal diameter of three tubes set as optimization variable and these variable are *D_tube_*_1_*,D_tube_*_2_ and *D_tube_*_3_. Its unit is millimeters.
[137]	Minimum energy consumption, uniform battery power depletion, and minimum delay	(8) $\mathrm{minimize}\;F\left(x\right)={\left(F_{1}\left(x\right),\ldots \ldots ,F_{n}\left(x\right)\right)}^{T})$	Where *F_i_*(*x*) is an objective function, for 1 ≤ *i* ≤ *n*; *w_i_* denotes a weight selected by a network designer to reflect the relative importance of the objective function; *c_i_* denotes a coefficient that not only scales *F_i_*(*x*) but also helps produce a one-dimensional function.
[138]	Maximizing the network lifetime subject to QoS constraints	(9) $$\begin{array}{l} \text{max}B_{s_{o}s_{d}}\left(℘\right)\\ \max L_{i}^{b} \end{array}$$	Multi-objective function. Objective 1 maximizes the residual energy of the selected nodes. Objective 2 maximizes the residual energy of the forwarding set. *B_s_o_s_d__* represents end-to-end path battery cost; ℘ represents the set of paths between the nodes; $L_{i}^{b}$ represents local battery cost for each node.

**Table 3:** Operation related objectives in wireless sensor networks.

**Ref.**	**Objectives**	**Equation**	**Details**
[85]	Maximizing the coverage rate, minimizing the percentage of active sensor nodes, and minimizing the unbalanced energy consumption	(10) $$\begin{array}{l} F_{1}=1-\frac{\left\lfloor \sum_{x\prime \in X}\sum_{y\prime \in Y}\right\rfloor \text{grid}\left(X\prime ,Y\prime \right)}{G}\\ F_{2}=\frac{\max\left(E_{i}\right)-\min\left(E_{i}\right)}{\max\left(E_{i}\right)}\\ F_{3}=\underset{j\in NC}{\text{∑}}\text{Statu}s_{j}\\ F=\arg\min\left\{F1,F2,F3\right\} \end{array}$$	Where G is total number of grid; *E_i_* is is the residual energy of sensor *i*; NC is total number of sensor in cluster; *Status_j_* represents the scheduling status of node *j*.
[86]	High network coverage, effective node utilization and more residual energy	(11) $$\begin{array}{l} \min\overset{n}{\underset{i=1}{\text{∑}}}\gamma_{i}\\ \max\bar{\omega }\left(T\right)\\ \min\alpha \times U\left(T\right)+\beta \times E\left(T\right) \end{array}$$	γ*_i_* is the decision variable; *ω̄*(*T*) represents the coverage degree of sensor networks; *U*(*T*) represents node utilization; *E*(*T*) represents the energy distribution of the network; *α* is the node utilization weighting coefficient; *β* is the energy balance weighting coefficient.
[89]	Minimization of the number of selected sensors and minimization of the information gap between the Fisher Information	(12) $\underset{\alpha }{\min}\left\{f_{1}\left(\alpha \right),f_{2}\left(\alpha \right),\ldots \ldots \ldots ,f_{n}\left(\alpha \right)\right\}$	Where *α* is the vector of decision variable with element *α_i_*.
[139]	Minimum Spanning Tree (MST)	(13) $\underset{xɛX}{\min}f\left(x\right)=\gamma_{\Delta }.a\left(x\right)+\gamma_{\chi }.b\left(x\right)+\gamma_{\theta }.c\left(x\right)$	*a*(*x*) is evaluation of delay along path *T_x_*; *b*(*x*) represents the evaluation of the co-channel Interference along the path in *T_x_; c*(*c*) is the evaluation of the link duration probability along the path in *T_x_*; γ_Δ_ is end-to-end delay weight; γ*_χ_* is co-channel Interference weight; γ*_θ_* represents link duration probability weight.
[140]	Maximizing the coverage rate, minimizing the percentage of active sensor nodes, and minimizing the unbalanced energy consumption	(14) $$\begin{array}{l} \min\left[f_{1}\left(x\right),f_{2}\left(x\right)\right]\\ \max f_{A}\left(x\right)=\frac{A_{s}}{A}\\ \min f_{1}\left(x\right)=1-f_{A}\left(x\right)\\ \min f_{2}\left(x\right)=\omega_{1}\rho +\omega_{2}E_{s} \end{array}$$	This is used for wireless sensor networks multi-objective coverage control model. Where *ω*_1_ is the energy consumption weight, and *ω*_2_ is the energy balance weight. *A_s_* represents the target area covered by the active nodes; *A* is the target region area; *E_s_* represents the balance level of energy consumption for the whole network; *ρ* represents the wireless sensor network node utilization.
[141]	Maximize the total throughput, minimize the total transmission power	(15) $$\begin{array}{c} \underset{ɛ,\rho_{\iota },\rho_{s}}{\min}F=w_{1}\left(1-F_{1}\right)+F_{2}w_{2}\\ F_{1}=\frac{\sum_{k=1}^{K}C_{k}}{\sum_{k=1}^{K}C_{k}^{\text{max}}}\\ F_{2}=\frac{E^{co_{2}}}{E_{\text{max}}^{CO}} \end{array}$$	Normalize the first objective between 0 and 1 and second objective is to reduce the carbon footprint. *C_k_* represents the channel capacity of the *k*th user for shared band; *C^m^ax_k_* represents upper bound of the sum-rate capacity *C*_k_; *E^CO^*^2^ is the *CO*_2_ emission; *w*_1_ and *w*_2_ are used to create a joint minimization (or maximization) objective.
[142]	Minimum interrogation cycle, maximum reader utilization, and energy efficiency	(16) $\underset{\left\{p_{i}^{c,s}\right\},\left\{\gamma_{i}^{c,s}\right\},S,W,E}{\min}S+\xi_{1}\left(W+\xi_{2}E\right)$	Three stage optimization to single stage optimization problem. *ξ*_1_ and *ξ*_2_ are constants which depend on the number of readers and the maximum output power; *W* represents the RFID reader utilization; *E* represents the RFID power consumption; *S* denotes the interrogation circle.
[143]	To balance network communication ability and energy efficiency	(17) $Q^{*}=\underset{a_{t}}{\max}Q_{i,t+1}\left(s_{t},p_{t}\right)$	Where Q* mean Q optimal value.
[144]	Operation	(18) $\underset{i=1}{\max}\sum_{i=1}^{N}b_{i}\text{and}\underset{i=1}{\min}\sum_{i=1}^{N}p_{i}$	Where *i*=1,2,3…,*N*; *b_i_* is the number of bits; *p_i_* is allocated power per subcarrier.

**Table 4:** Deployment related objectives in wireless sensor networks.

**Ref.**	**Objectives**	**Equation**	**Details**
[9]	Arrangement to maximize the area of coverage, minimize the net energy consumption, maximize the network lifetime, and minimize the number of deployed sensor nodes	(19) $\min Y=F\left(\vec{x}\right)=\left(\sum_{iɛs}e_{i},\sum_{jɛD}NC_{j}xH_{j}\right)$	*e_i_* represents the energy consumed at each node; *N* denotes number of sensor nodes; *NC_j_* is a non-coverage penalty parameter; *h_j_* is variable to indicate if demand point j is not covered; *D* represents set of demand points.
[108]	Maximize connectivity and minimize energy consumption of the network	(20) $E_{\text{min}}=\min\left[P_{1}+P_{2},\ldots .P_{j}+\ldots ..P_{N}\right]$	For *C_jH_* = 0, sensor *j* is disconnected. It means that sensor *C_jH_* is not within the scope of cover. *E_min_* is the minimum energy consumption; *P_j_* is the transmission power level of sensor *j*.
[111]	Optimal sensing, coverage and network lifetime	(21) $$\begin{array}{c} F_{\text{cov}}=\frac{\left[\sum_{x\prime =0}^{X}\sum_{y\prime =0}^{Y}g\left(x\prime ,y\prime \right)\right]}{\left(x\times y\right)}\\ F_{nt}=\text{Tim}e_{\text{last}}-\text{Tim}e_{\text{first}}\\ F_{\text{mov}}=\overset{N}{\underset{i=1}{\text{∑}}}\sqrt{{\left(x_{i}-x\right)}^{2}+{\left(y_{i}-y\right)}^{2}} \end{array}$$	First objective function is used for total sensing area, second objective function is used for network life and third objective function is used for moving cost of sensor nodes.
[112]	Coverage and lifetime	(22) $$\begin{array}{cc} \min & z\left(x\right)= \end{array}\left[z_{1}\left(x\right),z_{2}\left(x\right),\ldots ..,z_{M}\left(x\right)\right]$$	Vector fiunction *z* consisting of *M* objectives.
[128]	Maximize Coverage and Lifetime	(23) $$\begin{array}{c} \underset{\text{coverage}}{\max}f_{1}=\frac{U_{i=1,\ldots ,N}}{A}A_{i}\\ \underset{\text{lifetime}}{\max}f_{2}=\frac{T_{\text{failure}}}{T_{\text{max}}} \end{array}$$	*A_i_* is the area coved by *i^th^* node; *N* is total number of node and *A* is the area of region of interest.
[145]	Maximum coverage with minimum energy consumption	(24) $$\begin{array}{cc} \min & Y=F\left(\vec{x}\right)=\left(\sum_{iɛs}E_{i},\sum_{pɛM}NCov_{p}\right) \end{array}$$	*f*_1_ is the net energy consumed and 2nd objective function is maximize the area of coverage. *NCov_p_* are number of uncovered points, which are used to express coverage; *E_i_* is the energy consumed by each node *i*; *M* is the total number of monitoring points; *S* is the set of sensor nodes.
[146]	Number, position and orientation	(25) $F_{s}=\sum_{i=0}^{Nss}\left(w_{si}\times \varphi_{i}\right)$	Where 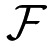 *_s_* is fitness function; *N_ss_* is maximum number of available sensors; *w_si_* is weight related to *i* simultaneously illuminated sensors; *φ_i_* is total angular interval with *i* illuminated sensors.
[147]	Bit-Error-Rate minimization, system throughput maximization, power consumption minimization	(26) $$\begin{array}{c} f_{\text{min}\_ber}=\frac{\sum_{i=1}^{N_{c}}1-\frac{\log_{10}\left(0.5\right)-\log_{10}\left(P_{bei}\right)}{\log_{10}\left(0.5\right)-\log_{10}\left(10^{-12}\right)}}{N_{c}}\\ f_{\text{max}\_tp}=\frac{\sum_{i=1}^{N_{c}}\frac{L_{i}}{L_{i}+O+H}.{\left(1-P_{bei}\right)}^{L_{i}+o}.R_{ci}.TDD_{i}}{N_{c}} \end{array}$$	First objective function for bit-error-rate and 2nd Object function for throughput. *N_c_* is number of carriers; *L_j_* is the size transmission frame size in bytes; *O* is physical layer overhead; *H* is MAC and IP layers overhead; *P_bei_* is the probability of bit error rate; *R_ci_* is the coding rate; *TDD* is percentage of transmit time.
[148]	Minimum number of sensor nodes and provide maximum coverage and connectivity	(27) $$\begin{array}{c} \min F_{1}=\overset{X\times Y}{\underset{i=1}{\text{∑}}}d_{i}\\ \min F_{2}=\overset{X\times Y}{\underset{i=1}{\text{∑}}}1-e^{-\left(R_{c}-R_{s}\right)} \end{array}$$	*R_c_* is the communication range of a node; *R_s_* is sensing range; *d_i_* represents the random deployment of sensor nodes; *X* and *Y* are the coordinates of a particular area.
